# Usefulness of the Population Health Metrics Research Consortium gold standard verbal autopsy data for general verbal autopsy methods

**DOI:** 10.1186/1741-7015-12-23

**Published:** 2014-02-04

**Authors:** Peter Byass

**Affiliations:** 1 WHO Collaborating Centre for Verbal Autopsy, Umeå Centre for Global Health Research, Umeå University, 90187 Umeå, Sweden; 2 School of Public Health, Faculty of Health Sciences, University of the Witwatersrand, Johannesburg, South Africa; 3 Epidemiology & Global Health, Department of Public Health and Clinical Medicine, Umeå University, 90187 Umeå, Sweden

**Keywords:** Verbal autopsy, Cause of death, Death registration, Low- and middle-income countries, InterVA

## Abstract

**Background:**

Verbal Autopsy (VA) is widely viewed as the only immediate strategy for registering cause of death in much of Africa and Asia, where routine physician certification of deaths is not widely practiced. VA involves a lay interview with family or friends after a death, to record essential details of the circumstances. These data can then be processed automatically to arrive at standardized cause of death information.

**Methods:**

The Population Health Metrics Research Consortium (PHMRC) undertook a study at six tertiary hospitals in low- and middle-income countries which documented over 12,000 deaths clinically and subsequently undertook VA interviews. This dataset, now in the public domain, was compared with the WHO 2012 VA standard and the InterVA-4 interpretative model.

**Results:**

The PHMRC data covered 70% of the WHO 2012 VA input indicators, and categorized cause of death according to PHMRC definitions. After eliminating some problematic or incomplete records, 11,984 VAs were compared. Some of the PHMRC cause definitions, such as ‘preterm delivery’, differed substantially from the International Classification of Diseases, version 10 equivalent. There were some appreciable inconsistencies between the hospital and VA data, including 20% of the hospital maternal deaths being described as non-pregnant in the VA data. A high proportion of VA cases (66%) reported respiratory symptoms, but only 18% of assigned hospital causes were respiratory-related. Despite these issues, the concordance correlation coefficient between hospital and InterVA-4 cause of death categories was 0.61.

**Conclusions:**

The PHMRC dataset is a valuable reference source for VA methods, but has to be interpreted with care. Inherently inconsistent cases should not be included when using these data to build other VA models. Conversely, models built from these data should be independently evaluated. It is important to distinguish between the internal and external validity of VA models. The effects of using tertiary hospital data, rather than the more usual application of VA to all-community deaths, are hard to evaluate. However, it would still be of value for VA method development to have further studies of population-based post-mortem examinations.

## Background

Verbal Autopsy (VA, the practice of interviewing witnesses of a death and processing the information into likely causes of death) is recognized as an important strategy for widening the scope of cause-specific mortality data, particularly in countries where most deaths pass unregistered and unattended by medical professionals. There has been considerable development in recent years of automated procedures for systematically coding VA data into cause of death outcomes, which are seen as a necessary strategy to underpin widespread, rapid and cost-effective approaches to registering cause of death [1].

Inevitably, one of the major challenges to developing fit-for-purpose VA interpretative models is capturing the necessary knowledge in a valid and usable manner. Some models have sought to be entirely based on data, including in some cases the application of machine learning techniques to construct the model’s evidence base [2,3]. Others have developed systematic approaches to incorporating human medical expertise as the basis of model building [4]. In both cases, there is also an obvious desire to be able to test models with high-quality VA data in order to demonstrate validity in terms of relationships between the input side (VA interview material) and the output side (cause of death assignment) [5,6].

To meet this need, the Population Health Metrics Research Consortium (PHMRC) undertook a data collection exercise between 2007 and 2010 in four countries (India, Mexico, Tanzania and the Philippines) which documented 12,542 deaths in high-level hospitals where excellent diagnostic facilities were available during final illnesses, and then followed up the deaths with VA interviews [7]. This dataset was dubbed as ‘gold standard’ VA data in findings published in 2011. In October 2013 the dataset was released into the public domain, and this dataset is examined here in terms of its compatibility with the WHO 2012 VA standard [8,9], its completeness and quality, and consistency between the hospital cause of death categorization and the InterVA-4 model (which is exactly compatible with the WHO 2012 VA standard) [10]. The objectives in undertaking these analyses are to arrive at a better understanding of how useful the PHMRC dataset might be for developing general VA methods and to explore the external validity of the dataset.

## Methods

The PHMRC public-domain dataset [11] contains tertiary hospital assignments of cause of death and responses to subsequent VA interviews. The data are divided into three sections according to variations in the VA instrument used for the neonatal period (including stillbirths), childhood (one month to eleven years) and adulthood (twelve years and older). Two separate sites were covered in India (Andra Pradesh and Uttar Pradesh) and Tanzania (Dar es Salaam and Pemba island), with one each in the Philippines (Bohol) and Mexico (Mexico City). The public domain dataset contains slightly fewer cases than were originally reported (12,530 versus 12,542). An overall summary of the data is presented in Table 1 by site, age group and cause of death according to the hospital diagnosis. No hospital data other than the cause of death category are available in the dataset, and the cause categories were pre-defined as previously described [7].

**Table 1:** Summary of PHMRC VA dataset for 12,530 deaths by cause of death, age group (adult, child and neonate) and site

**Site**	**Andhra Pradesh**			**Bohol**			**Dar es Salaam**			**Mexico City**			**Pemba**			**Uttar Pradesh**			**Total**
**Cause of death category**	**Ad**	**Ch**	**Ne**	**Ad**	**Ch**	**Ne**	**Ad**	**Ch**	**Ne**	**Ad**	**Ch**	**Ne**	**Ad**	**Ch**	**Ne**	**Ad**	**Ch**	**Ne**	
AIDS	136	1					203	19		120						43			522
Acute myocardial infarction	101			116			3			76						104			400
Asthma	21			6			12			1			6			1			47
Birth asphyxia			115			50			134			45			66			52	462
Bite of venomous animal	31	33		1						1						33	21		120
Breast cancer	3			28			90			69						5			195
COPD	25			4			2			63						77			171
Cervical cancer	3			9			108			31						4			155
Cirrhosis	51			39			27			133						63			313
Colorectal cancer	7			24			33			35									99
Congenital malformation			63			41			62			61			19			3	249
Diabetes	88			77			59			105			38			47			414
Diarrhea/dysentery	77	35		27	22		37	50		18	8		21	64		48	77		484
Drowning	32	29		4			9	13			4		28	6		33	31		189
Encephalitis		7															34		41
Epilepsy	30			5			6			2						5			48
Esophageal cancer	1			5			34												40
Falls	32	12		38	6		11	1		36	2		26	4		30	24		222
Fires	35	30		7			25	15		18	1		6	7		31	15		190
Hemorrhagic fever		30			13			1									7		51
Homicide	31			35			30			37			3			31			167
Leukemia/lymphomas	7			15			59			72						3			156
Lung cancer	5			23			11			59						8			106
Malaria	29	12					34	102					2	2		35			216
Maternal	71			41			135			39			46			136			468
Measles					1												22		23
Meningitis		6			2			23									27		58
Meningitis/sepsis			2			19			132			7			1			5	166
Other cancers		7			8			2			3						8		28
Other cardiovascular diseases	76	14		78	12		112	32		81	7					69	11		492
Other defined causes of child deaths		31			20			31			48			30			34		194
Other digestive diseases		5			18			9			13			3					48
Other infectious diseases	34	4		51	9		37	9		37	6		3	2		101	37		330
Other injuries	31			7			23			2			3			37			103
Other non-communicable diseases	90			125			171			122			54			37			599
Pneumonia	55	102	7	142	124	18	95	111	43	102	22		41	134	2	105	39	13	1155
Poisonings	30	3		3	3		8	2		9			1	1		35	9		104
Preterm delivery			85			90			244			127			53			61	660
Prostate cancer	1			4			32			11									48
Renal failure	102			68			49			92						105			416
Road traffic	39	33		55	3		31	15		34	2		11	8		32	31		294
Sepsis		29			21			32			10						46		138
Stillbirth			102			156			435			75			119			118	1005
Stomach cancer	5			5			21			31									62
Stroke	125			179			103			122						101			630
Suicide	49			16			13			11			2			33			124
TB	101			22			103			17			6			27			276
Violent death		26															26		52
ALL CAUSES	1,554	449	374	1,259	262	374	1,726	467	1,050	1,586	126	315	297	261	260	1,419	499	252	12,530

Ad, adult; Ch, children; COPD, chronic obstructive pulmonary disease; Ne, neonate; PHMRC, Population Health Metrics Research Consortium; TB, tuberculosis; VA, verbal autopsy.

The VAs that were subsequently performed on the PHMRC hospital deaths used a set of standard forms covering each of the three age groups, yielding databases with structures defined in a spreadsheet [12]. In addition, the original VAs contained an open narrative section which is not available in the public domain dataset, but the dataset does include some machine-extracted variables indicating the presence of certain key words within those narratives. However, in the present analyses it was not possible to make use of those key word variables because no clear distinction was made between narratives containing, for example, the word ‘heartbeat’ in the phrases ‘the baby’s heartbeat was normal’ or ‘the baby had no heartbeat’.

The WHO 2012 VA standard [8] and InterVA-4 [10] define 244 yes/no indicators covering VAs relating to various age and sex groups. A correspondence table between the WHO 2012 indicators and the PHMRC database is available in Additional file 1. Overall 170/244 (69.7%) of the WHO indicators were available in the PHMRC dataset, and these variables were extracted into a dataset for processing using the InterVA-4 model (version 4.02) [13]. Some problems were encountered in the age and sex variables in the PHMRC dataset. Dates of birth and death were recorded in two duplicate sets of variables (g1_01/6 and g5_01/3) as well as duplicate variables for age at death (g1_07 and g5_04). For some individuals there were inconsistencies between these age-related variables, and cases were dropped where these inconsistencies resulted in ambiguities between WHO 2012 age groups. Overall, 494 cases (3.9%) in the PHMRC dataset had no valid age or sex recorded and were excluded from InterVA-4 processing. A further 36 cases (0.3%) reported none of the WHO 2012 symptom indicators and were likewise excluded. Analyses of InterVA-4 outputs are, therefore, based on 11,984 cases.

The InterVA-4 model requires estimates of HIV/AIDS and malaria levels among deaths in what would normally be a population (rather than a group of hospital cases), as described in the User Guide [14]. In the PHMRC study, there was no intention that the deaths recorded were in any way representative of local populations, and so these parameters were set according to the overall levels of HIV/AIDS and malaria according to the hospital causes of death. In Andhra Pradesh, Dar es Salaam and Uttar Pradesh malaria levels were high (>1% of all deaths) while in the remaining sites malaria levels were very low (<0.01%). Similarly for HIV/AIDS, in Bohol and Pemba HIV/AIDS levels were very low (<0.01%), while in the remaining sites the level was high (>1%). InterVA-4 generates from zero to three most likely causes per case, each with an associated likelihood; cases with zero causes reflect complete uncertainty. The sum of the likelihoods for each case was always taken to be one in these analyses, including, where necessary, an indeterminate cause component.

The WHO 2012 VA standard defines 62 cause of death categories (including stillbirths) with corresponding International Classification of Diseases, 10^th^ revision (ICD-10) codes. These differ in some respects from the PHMRC cause categories, and for the purposes of comparison the correspondence between the WHO and PHMRC categories (34 cause version as previously published [7]) is shown in Table 2. The concordance correlation coefficient between cause-specific mortality fractions (CSMF) was calculated using Stata, according to the method described by Lin [15].

**Table 2:** Working equivalence between WHO 2012 and PHMRC cause of death categories

**WHO 2012 VA cause categories**	**PHMRC cause categories**
01.01 Sepsis (non-obstetric)	Sepsis
01.02 Acute resp infect incl pneumonia	Pneumonia
01.03 HIV/AIDS related death	AIDS
01.04 Diarrhoeal diseases	Diarrhea/dysentery
01.05 Malaria	Malaria
01.06 Measles	Measles
01.07 Meningitis and encephalitis	Meningitis; encephalitis
01.08 & 10.05 Tetanus	Other infectious diseases
01.09 Pulmonary tuberculosis	TB
01.10 Pertussis	Other infectious diseases
01.11 Haemorrhagic fever	Hemorrhagic fever
01.99 Other and unspecified infect dis	Other infectious diseases
02.01 Oral neoplasms	Other cancers
02.02 Digestive neoplasms	Esophageal cancer; stomach cancer
02.03 Respiratory neoplasms	Lung cancer
02.04 Breast neoplasms	Breast cancer
02.05 & 02.06 Reproductive neoplasms M/F	Cervical cancer; prostate cancer
02.99 Other and unspecified neoplasms	Leukemia/lymphomas; other cancers
03.01 Severe anaemia	Other non-communicable diseases
03.02 Severe malnutrition	Other non-communicable diseases
03.03 Diabetes mellitus	Diabetes
04.01 Acute cardiac disease	Acute myocardial infarction
04.02 Stroke	Stroke
04.03 Sickle cell with crisis	Other non-communicable diseases
04.99 Other and unspecified cardiac dis	Other cardiovascular diseases
05.01 Chronic obstructive pulmonary dis	COPD
05.02 Asthma	Asthma
06.01 Acute abdomen	Other digestive diseases; other non-communicable diseases
06.02 Liver cirrhosis	Liver cirrhosis
07.01 Renal failure	Renal failure
08.01 Epilepsy	Epilepsy
98 Other and unspecified NCD	Other digestive diseases; other non-communicable diseases
10.06 Congenital malformation	Congenital malformation
10.01 Prematurity	Preterm delivery
10.02 Birth asphyxia	Birth asphyxia
10.03 Neonatal pneumonia	Pneumonia
10.04 Neonatal sepsis	Meningitis/sepsis
10.99 Other and unspecified neonatal CoD	n/a
11.01 Fresh stillbirth	Stillbirth
11.02 Macerated stillbirth	Stillbirth
12.01 Road traffic accident	Road traffic
12.02 Other transport accident	Other injuries
12.03 Accid fall	Falls
12.04 Accid drowning and submersion	Drowning
12.05 Accid expos to smoke fire & flame	Fires
12.06 Contact with venomous plant/animal	Bite of venomous animal
12.10 Exposure to force of nature	Other injuries
12.07 Accid poisoning & noxious subs	Poisonings
12.08 Intentional self-harm	Suicide
12.09 Assault	Homicide; violent death
12.99 Other and unspecified external CoD	Other injuries
09.01 Ectopic pregnancy	Maternal
09.02 Abortion-related death	Maternal
09.03 Pregnancy-induced hypertension	Maternal
09.04 Obstetric haemorrhage	Maternal
09.05 Obstructed labour	Maternal
09.06 Pregnancy-related sepsis	Maternal
09.07 Anaemia of pregnancy	Maternal
09.08 Ruptured uterus	Maternal
09.99 Other and unspecified maternal CoD	Maternal
99 Indeterminate	Other defined causes of child deaths

COPD, chronic obstructive pulmonary disease; PHMRC, Population Health Metrics Research Consortium; TB, tuberculosis; VA, verbal autopsy.

A separate experiment was carried out using the DHS 2010 Afghanistan national mortality survey [16] database as an independent source, to estimate the effect of losing the missing PHMRC VA variables on the overall mortality cause distribution as determined by InterVA-4. This involved running InterVA-4 separately on a dataset extracted from the DHS and then re-running with the non-PHMRC indicators removed.

No specific ethical clearance was required for this study, since it only used the public domain anonymized PHMRC VA dataset, the public domain DHS 2010 Afghanistan dataset and the public domain InterVA-4 model.

## Results

From the overall 12,530 cases in the PHMRC VA dataset, 11,984 cases met the basic minimum requirements of the WHO 2012 VA standard (valid age, sex and some symptom data). Among these cases, 1,688 (14.1%) of the PHMRC causes of death fell into categories described as ‘Other…’ which do not represent specific causes of death.

**Figure 1:**
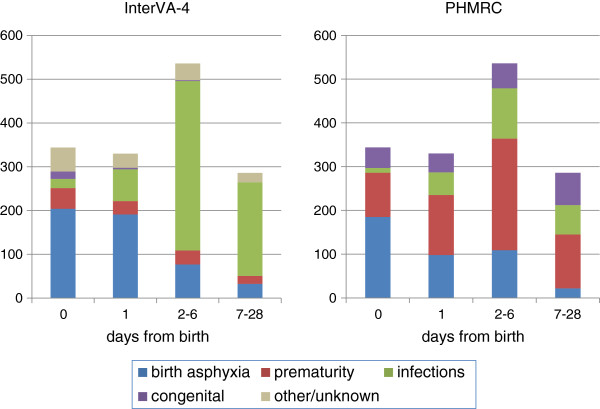
**Neonatal deaths by cause and time of death (n = 1,496) according to InterVA-4 and PHMRC cause of death assignments.** PHMRC, Population Health Metrics Research Consortium.

Deaths among women aged 12 to 49 accounted for 1,884/11,984 (15.7%) of the cases, and 458 (24.3%) of these deaths were classified as ‘Maternal’ in the PHMRC dataset. However, 17/1,884 (0.9%) of these deaths had contradictory pregnancy status information in the VA data (for example, both pregnant and recently delivered at death). Of the 458 PHMRC ‘Maternal’ deaths, 90 (19.7%) were not reported in the VA interview to have been pregnant at or within six weeks of death. Hospital diagnoses reported 1,603 neonatal deaths, 107 (6.7%) of which were said to be stillbirths in the VA interviews. The remaining 1,496 neonatal deaths are presented by cause and time in Figure 1. In 147/1,001 (14.7%) deaths which were recorded as stillbirths in hospitals, the circumstances were recorded as neonatal deaths in VA interviews, mostly during the first day of life. However, the symptoms reported in many of these discrepant cases were contradictory, leading to many being categorized as indeterminate in cause by InterVA-4.

A high proportion of cases (7,898/11,984; 65.9%) were reported as having respiratory symptoms in the VA interviews, although 2,134/11,984 (17.8%) had respiratory-related PHMRC causes of death assigned. Table 3 shows the proportions of cases reporting various respiratory symptoms for respiratory-related PHMRC causes (2,134/11,984; 17.8%; comprising asthma, birth asphyxia, COPD, lung cancer, pneumonia, TB) and for other PHMRC causes of death.

**Table 3:** Numbers (%) of verbal autopsy interviews reporting respiratory symptoms for respiratory-related and other PHMRC causes of death, by age group

**Symptom**	**n 11,984**	**Respiratory-related causes of death**^**a**^			**Other causes of death**		
		**Adults (n = 1,074)**	**Children (n = 519)**	**Neonates (n = 541)**	**Adults (n = 6,338)**	**Children (n = 1,449)**	**Neonates (n = 2,063)**
Any breathing problem	7,898	945 (88.0%)	465 (89.6%)	435 (80.4%)	4,260 (67.2%)	906 (62.5%)	887 (43.0%)
Rapid breathing	4,126	578 (53.8%)	320 (61.7%)	122 (22.6%)	2,312 (36.5%)	511 (35.3%)	283 (13.7%)
Breathless on exertion	415	96 (8.9%)			319 (5.0%)		
Breathless lying down	1,965	322 (30.0%)			1,643 (25.9%)		
Chest indrawing	987		270 (52.0%)	101 (18.7%)		349 (24.1%)	267 (12.9%)
Wheezing or grunting	2,188	323 (30.0%)	240 (46.2%)	60 (11.1%)	1,018 (16.1%)	340 (23.5%)	207 (10.0%)

^a^Includes asthma, birth asphyxia, chronic obstructive pulmonary disease, lung cancer, pneumonia and tuberculosis. PHMRC, Population Health Metrics Research Consortium.

**Figure 2:**
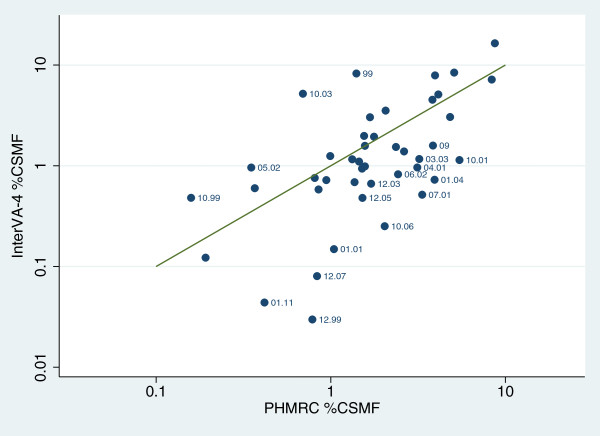
**Correlation between cause-specific mortality fractions as determined by PHMRC hospital data and the InterVA-4 model for 11,984 verbal autopsy cases in 43 cause categories.** Data points are labelled with the WHO 2012 VA cause of death category codes and shown against the line of equivalence. PHMRC, Population Health Metrics Research Consortium.

CSMFs are shown in Table 4 by age group and for all ages combined for 43 cause-of-death categories as determined by InterVA-4 and PHMRC. Differences in CSMF and 95% confidence intervals (CIs) of those differences are shown for the all-age CSMFs. For 22/43 causes (51.1%), CSMF differences were less than 1%. Figure 2 illustrates the correlation between PHMRC and InterVA-4 CSMFs in the 11,984 cases and 43 cause categories analyzed. The concordance correlation coefficient was 0.612 (95% CI 0.454 to 0.770). Excluding the residual categories, the concordance correlation coefficient increased to 0.665 (95% CI 0.502 to 0.827).

**Table 4:** Cause-specific mortality fractions (%) for 11,984 deaths by age group and for all ages as determined by InterVA-4 and PHMRC hospital causes of death

**Cause of death category**	**Adults**		**Children**		**Neonates**		**Stillbirths**		**All ages**			
	**InterVA**	**PHMRC**	**InterVA**	**PHMRC**	**InterVA**	**PHMRC**	**InterVA**	**PHMRC**	**InterVA**	**PHMRC**	**diff**	**95% CI**
01.01 Sepsis (non-obstetric)	0.13		0.4	6.35					0.15	1.04	−0.89	−1.09 to −0.70
01.02 Acute resp infect, incl pneumonia	14.97	7.07	43.78	26.37					16.45	8.70	7.74	6.91 to 8.58
01.03 HIV/AIDS related death	6.57	6.44	6.34	0.91					5.11	4.13	0.97	0.44 to 1.51
01.04 Diarrheal diseases	0.17	2.98	3.78	12.7					0.73	3.93	−3.20	−3.58 to −2.82
01.05 Malaria	1.8	1.23	11.68	5.59					3.03	1.68	1.35	0.97 to 1.74
01.06 Measles	0.03		0.64	1.17					0.12	0.19	−0.07	−0.17 to 0.03
01.07 Meningitis and encephalitis	0.76		1.73	4.93					0.82	0.81	0.01	−0.22 to 0.23
01.09 Pulmonary tuberculosis	5.64	3.33	0.24						3.53	2.06	1.47	1.05 to 1.89
01.11 Hemorrhagic fever			0.27	2.54					0.04	0.42	−0.37	−0.49 to −0.25
01.99 Other and unspecified infect dis	1.89	3.35	1.33	3.4					1.39	2.63	−1.24	−1.60 to −0.89
02.02 Digestive neoplasms	3.2	2.51							1.98	1.55	0.43	0.09 to 0.76
02.03 Respiratory neoplasms	0.94	1.38							0.58	0.85	−0.27	−0.48 to −0.06
02.04 Breast neoplasms	1.59	2.54							0.99	1.57	−0.58	−0.87 to −0.30
02.05, 02.06 Reproductive neoplasms M,F	2.55	2.54							1.58	1.57	0.01	−0.31 to 0.32
02.99 Other and unspecified neoplasms	1.78	1.98		1.37					1.10	1.45	−0.35	−0.63 to −0.07
03.03 Diabetes mellitus	1.88	5.19	0.03						1.17	3.21	−2.05	−2.41 to −1.68
04.01 Acute cardiac disease	1.55	5.06							0.96	3.13	−2.17	−2.52 to −1.81
04.02 Stroke	4.93	7.8							3.05	4.82	−1.77	−2.27 to −1.28
04.99 Other and unspecified cardiac dis	12.75	5.38	0.03	3.81					7.89	3.96	3.94	3.34 to 4.53
05.01 Chronic obstructive pulmonary dis	1.87	2.15							1.16	1.33	−0.17	−0.45 to 0.11
05.02 Asthma	1.5	0.57	0.22						0.96	0.35	0.61	0.41 to 0.81
06.02 Liver cirrhosis	1.33	3.93							0.82	2.43	−1.61	−1.92 to −1.29
07.01 Renal failure	0.83	5.4							0.52	3.34	−2.82	−3.17 to −2.48
08.01 Epilepsy	0.87	0.59	0.37						0.60	0.37	0.23	0.06 to 0.41
98 Other and unspecified NCD	12.13	7.61	5.59	2.34					8.42	5.09	3.33	2.69 to 3.96
09 Maternal CoD	2.57	6.22							1.59	3.85	−2.26	−2.67 to −1.85
10.01 Prematurity					7.99	40.8	0.85		1.14	5.46	−4.32	−4.77 to −3.87
10.02 Birth asphyxia					31.46	28.57	3.79		4.52	3.82	0.70	0.20 to 1.21
10.03 Neonatal pneumonia					38.47	5.18	0.54		5.19	0.69	4.50	4.08 to 4.92
10.04 Neonatal sepsis					5.03	10.23	0.18		0.63	1.37	−0.74	−0.99 to −0.49
10.06 Congenital malformation					1.45	15.22	0.68		0.25	2.04	−1.78	−2.05 to −1.52
10.99 Other and unspecified neonatal CoD					2.77		1.33		0.48	0.00	0.48	0.35 to 0.60
11 Stillbirth					5.62		76.94	100	7.18	8.35	−1.17	−1.85 to −0.50
12.01 Road traffic accident	1.51	2.6	3.68	4.57					1.54	2.36	−0.82	−1.17 to −0.47
12.03 Accid fall	0.7	2.17	1.41	2.18					0.66	1.70	−1.04	−1.31 to −0.77
12.04 Accid drowning and submersion	0.57	1.39	3.55	3.96					0.94	1.51	−0.57	−0.85 to −0.30
12.05 Accid expos to smoke, fire & flame	0.37	1.59	1.54	3.25					0.48	1.52	−1.04	−1.29 to −0.79
12.06 Contact with venomous plant/animal	0.55	0.84	2.31	2.59					0.72	0.94	−0.22	−0.45 to 0.01
12.07 Accid poisoning and noxious subs	0.04	1.11	0.33	0.91					0.08	0.83	−0.75	−0.92 to −0.58
12.08 Intentional self-harm	2.01	1.61	0.03						1.25	0.99	0.26	−0.01 to 0.52
12.09 Assault	2.27	2.2	3.3	2.49					1.94	1.77	0.18	−0.17 to 0.52
12.99 Other and unspecified external CoD	0.02	1.27	0.12						0.03	0.78	−0.75	−0.92 to −0.59
99 Indeterminate	7.71		7.31	8.54	7.21		15.69		8.24	1.40	6.84	6.31 to 7.38

CI, confidence interval; PHMRC, Population Health Metrics Research Consortium.

In an empirical comparison of the DHS 2010 Afghanistan dataset (3,349 deaths) run through InterVA-4 with and without the input indicators not available in the PHMRC dataset, overall 16.2% of causes of death were re-assigned due to the missing indicators. There was a net shift of 2.5% of all deaths into residual categories. Specific causes which increased most appreciably due to the missing indicators included neonatal pneumonia, acute abdomen and asthma, while the greatest decreases due to the missing indicators were seen in birth asphyxia, COPD and diarrheal diseases. However, the top six ranked causes of death were the same in both cases.

## Discussion

The PHMRC VA dataset undoubtedly represents an important attempt to collect a set of reference data for VA that have corresponding causes of death established in high-level facilities offering good diagnostic procedures. Obviously, since the commencement of the PHMRC study preceded the WHO 2012 VA standard, the dataset could not correspond directly to that standard. In the analyses presented here, equivalence between the PHMRC and WHO 2012 VA cause of death categories and VA indicators has been established as transparently and completely as possible, given the inherent differences. Empirical findings from the Afghan data comparison suggest that the WHO 2012 indicators which are missing from the PHMRC dataset may have some effect in terms of decreased certainty of cause attribution and redistribution of certain causes, although the overall cause of death profile may not change markedly in consequence. The relatively robust nature of the InterVA model has also been demonstrated in a previous sensitivity analysis [17].

Nevertheless, there are some differences both of principle and detail that cannot be totally reconciled between the PHMRC and WHO 2012 approaches. The PHMRC study set out to rigorously define causes of death, but this involved imposing some hierarchies which may not be universally recognized and are not always consistent with ICD-10 coding. For example, prematurity as cause of death can be applied according to the PHMRC definitions if a baby is born at <33 weeks gestational age and experienced ‘death from another medically documented neonatal condition’ irrespective of that condition [7]. This differs appreciably from ICD-10 coding: ‘The mode of death, e.g. heart failure, asphyxia or anoxia, should not be entered… [as the most important cause] …unless it was the only fetal or infant condition known. This also holds true for prematurity’. [18]. As can be seen from the PHMRC causes of death in Figure 1, prematurity is consequently the dominant component of mortality throughout the neonatal period. InterVA-4, on the other hand, ascribes more of these later neonatal deaths among premature babies to infectious causes, based on their subsequently reported symptoms. Neither of these two approaches is necessarily right or wrong, and both may actually be different views of the same reality; but the differences are very important in statistical descriptions of neonatal mortality and in assessing the comparability of different methods of cause of death ascertainment.

Unfortunately, the PHMRC public-domain dataset does not include any clinical details from the hospital work-ups in addition to the assigned cause of death. It would add considerable value to have basic clinical parameters for these deaths irrespective of cause of death. As has been shown in a multicenter analysis of VA cause of death against HIV serostatus, being infected with HIV has consequences for cause of death far beyond the number of classic AIDS deaths [19], and this may also be true for other factors routinely recorded as clinical parameters, such as malaria parasitemia, hemoglobin levels, and so on. It would also be of interest to have included the PHMRC local physicians’ interpretation of the VAs in order to see how those related to VA data, even though the PHMRC group previously concluded that physicians did not perform particularly well against the hospital causes of death [20].

The very high proportion of VA interviews reporting respiratory symptoms in the PHMRC dataset is also worth noting. Table 3 shows rather non-specific relationships between respiratory symptoms and respiratory causes of death in the PHMRC data, which need further investigation and explanation. One possibility is that many patients in tertiary facilities may receive oxygen therapy in the final stages of an illness leading to death, which may be interpreted as respiratory difficulties by family and friends when later responding to a VA interview. In any case, the high proportion of VAs reporting respiratory symptoms leads InterVA-4 to assign high fractions of respiratory-related causes of death. Again, further clinical details in terms of treatment received in the final illness would add value to the utility of the PHMRC dataset. By extension, this and similar issues also raise questions of the validity of using tertiary facility deaths as the evidence base for VA methods. For example, since all the injury cases in the PHMRC data were hospitalized, clearly none of them could have been instantaneous or near-instantaneous fatalities. On closer examination, many of the VAs for injury cases also reported a range of symptoms not obviously associated with their injuries by the time they died in hospital; but, according to the PHMRC protocols, any ‘third-party written account’ of an injury makes that injury the cause of death, irrespective of further clinical details [7].

There are some significant omissions in the PHMRC indicators compared with the WHO 2012 standard. Many of the detailed items relating to obstetric causes of death were missing from the PHMRC instrument, for example. Other items were recorded more precisely by PHMRC, such as details around loss of consciousness and tobacco consumption. Inevitably the comparability of cause of death between the PHMRC cause categories and the WHO 2012 VA cause categories generated by InterVA-4 are somewhat compromised by the differences in cause definitions and by missing 30% of the WHO 2012 VA indicators. Given these sources of variation, the overall concordance correlation coefficient of 0.6, as reflected in Figure 2 for specific cause categories, reflected reasonably good agreement. Some particular cause categories, such as pneumonia, some neonatal causes and certain residual groupings, accounted for much of the non-concordance.

Unsurprisingly, there were cases within the PHMRC database which showed blatant inconsistencies between hospital cause of death and responses to some VA questions. The most obvious were the 147 cases recorded as stillbirths in hospital but which were described as neonatal deaths in VA interviews, and the 90 hospital maternal deaths described as neither pregnant nor recently delivered in VA interviews. There is no way of knowing from the dataset whether these reflected data quality issues either in the hospital causes of death or VA material, or whether VA respondents were actually, either knowingly or unwittingly, presenting a different picture. It is, of course, likely that there were similar phenomena relating to other parameters which cannot be so easily checked. However, it raises an important issue if such a dataset is to be used to build an evidence-based model for VA interpretation, because any data-driven model based solely on these data would ‘learn’, for example, that a proportion of stillbirths show signs of life and that a proportion of maternal deaths occur in non-pregnant women. These effects are of particular concern in the case of, for example, the random forest and tariff models which the PHMRC group has built exclusively from this dataset and then evaluated using the same data [2,3]. Earlier work with artificial neural networks showed that VA models could be built with extremely high internal validity but had very limited external validity [21]. PHMRC’s earlier conclusions that independent methods (physician interpretation [20] and InterVA-3 [22]) performed less well against the PHMRC dataset than their internally-derived methods essentially confirm that the internal and external validities of automated VA methods can be very different. These findings are also reflected in a systematic review of automated VA methods [23]. There may be an argument for an expert panel to censor unreliable or contradictory cases from any VA reference database before using it to build VA models, in order to improve external validity. In addition, including residual cause of death categories in data for building models is unlikely to be very productive, given that residual categories are likely to include multifarious symptomatology, with no clear mapping between symptoms and cause of death categories.

## Conclusions

The PHMRC VA dataset is a unique resource in terms of offering mappings from causes of death determined in tertiary hospitals to corresponding VA data. The dataset would be all the more valuable if it included clinical diagnostic data, details of hospital treatment and local physicians’ interpretation of the VA material. In addition, a more usable presentation of terms included in the VA narratives might add further insights.

Such a dataset basically has two potential uses in relation to VA methods in general. Firstly, it is potentially a source of reference VA data which can be used to evaluate various approaches intended for interpreting other VA material, basically as done here in Figure 2. When used in that way, it should not be applied to data-driven models that have themselves been built from this same reference dataset to avoid confusion between the internal and external validity of models. Secondly, it is potentially a source of evidence between established causes of death and VA data that can be used to build evidence-based models for interpreting other VA data. Used in this context, it would be important to censor obviously misleading cases and probably also to exclude residual cause of death categories before extracting evidence from the dataset into models. In both uses, it is impossible to completely discount the effect of taking only tertiary hospital cases and cause of death assignments, which clearly differ in some important respects from more usual all-population applications of VA. Nevertheless, this effect has to be weighed against the advantages of having hospital causes of death based on sound clinical evidence.

It would still be very interesting to see studies of all-population post-mortem series from settings in low- and middle-income countries in order to build a more definitive evidence base from which to construct and validate VA methods [24].

## Competing interests

The author declares that he has no competing interests, and points out that the InterVA models which he has developed are entirely public domain assets.

## Supplementary Material

Additional file 1 Correspondence between WHO 2012 verbal autopsy indicators and Population Health Metrics Research Consortium variables. Click here for file
