# Supplementary material for: Usefulness of the Population Health Metrics Research Consortium gold standard verbal autopsy data for general verbal autopsy methods
Source: BMC Med. 2014 Feb 4;12:23. doi: 10.1186/1741-7015-12-23 (PMC3912496; doi:10.1186/1741-7015-12-23)
Supplement: Additional file 1 — Correspondence between WHO 2012 verbal autopsy indicators and Population Health Metrics Research Consortium variables. [file 1741-7015-12-23-S1.pdf]

WHO 2012 VA code	WHO 2012 InterVA-4 indicator	Equivalent PHMRC variable(s) (IHME_PHMRC_VA_DATA_CODEBOOK_Y2013M09D11_0.xlsx)
1A110A	Was the deceased male?	g1_05
1A110B	Was the deceased female?	
1A260A	Was (s)he aged 65 years or over at death?	g1_01d; g1_01m; g1_01y; g1_06d; g1_06m; g1_06y; g5_01d; g5_01m; g5_01y; g5_03d; g5_03m; g5_03y; g1_07a; g1_07b; g1_07c; g5_04a; g5_04b; g5_04c
1A260B	Was (s)he aged 50 to 64 years at death?	
1A260C	Was (s)he aged 15-49 years at death?	
1A260D	Was (s)he aged 5 to 14 years at death?	
1A260E	Was (s)he aged 1 to 4 years at death?	
1A260F	Was (s)he aged 1 to 11 months at death?	
1A260G	Was (s)he aged 28 days or less at death?	
1A260H	Did the baby die within 24 hours of birth?	
1A260I	Did baby die more than 24 hours after but less than 48 hours from birth?	
1A260J	Did baby die more than 48 hours from birth but within the first week of life?	
1A260K	Did baby die after first week, but within first 28 days?	
1A260L	Was this a woman aged 12 to 19 years at death?	
1A260M	Was this a woman aged 20-34 years at death?	
1A260N	Was this a woman aged 35-49 years at death?	
3A100	Was there any diagnosis of Tuberculosis?	a1_01_4
3A110	Was there any diagnosis of HIV/AIDS?	a1_01_14
3A120	Did (s)he have a recent positive test for malaria?	not available
3A130	Did (s)he have a recent negative test for malaria?	not available
3A140	Was there any diagnosis of Measles?	not available
3A150	Was there any diagnosis of High Blood pressure?	a1_01_10
3A160	Was there any diagnosis of heart disease?	a1_01_9
3A170	Was there any diagnosis of diabetes?	a1_01_7
3A180	Was there any diagnosis of asthma?	a1_01_1
3A190	Was there any diagnosis of epilepsy?	a1_01_8
3A200	Was there any diagnosis of cancer?	a1_01_3
3A210	Was there any diagnosis of Chronic Obstructive Pulmonary Disease (COPD)?	a1_01_13

WHO 2012 VA code	WHO 2012 InterVA-4 indicator	Equivalent PHMRC variable(s) (IHME_PHMRC_VA_DATA_CODEBOOK_Y2013M09D11_0.xlsx)
3A220	Was there any diagnosis of dementia?	a1_01_5
3A230	Was there any diagnosis of depression?	a1_01_6
3A240	Was there any diagnosis of stroke?	a1_01_12
3A250	Was there any diagnosis of sickle cell disease?	not available
3A260	Was there any diagnosis of kidney disease?	not available
3A270	Was there any diagnosis of liver disease?	not available
3A280	Did (s)he die during the wet season?	not available
3A290	Did (s)he die during the dry season?	not available
3A300A	Was (s)he ill for less than 3 weeks before death?	a2_01 < 21; c1_21 < 21
3A300B	Was (s)he ill for 3 weeks or more before death?	a2_01 >= 21; c1_21 >= 21
3A310	Did (s)he die suddenly?	a2_01 <= 1; c1_21=0 & c1_26 <> "Stillbirth"; c3_49
3B100	Did (s)he have a fever?	a2_02; a2_05 & a2_06; c3_26; c4_01
3B110A	Did (s)he have fever for less than 2 weeks before death?	fever & a2_03 < 14; c3_26 & c3_28 < 14; c4_01 & c4_02 < 14
3B110B	Did (s)he have fever for 2 weeks or more before death?	fever & a2_03 >= 14; c3_26 & c3_28 >=14; c4_01 & c4_02 >= 14
3B120	Did (s)he have night sweats?	a2_06; c4_05 = "Only at night"
3B130	Did (s)he have a cough?	a2_32; c4_12
3B140A	Did (s)he have a cough for less than 3 weeks before death?	a2_32 & a2_33 < 14; c4_12 & c4_13 < 14
3B140B	Did (s)he have a cough for 3 weeks or more before death?	a2_32 & a2_33 >= 14; c4_12 & c4_13 >= 14
3B150	Was the cough productive, with sputum?	a2_32 & a2_34
3B160	Did (s)he cough up blood?	a2_32 & a2_35
3B170	Did (s)he make a whooping sound when coughing?	not available
3B180	Did (s)he have any breathing problem?	a2_36; a2_40; a2_42; c3_05; c3_17; c4_16
3B190	Did (s)he have fast breathing?	a2_40; c3_20; c4_18
3B200A	Did (s)he have fast breathing for less than 2 weeks?	a2_40 & a2_41 < 14; c3_20 & c3_21 < 14; c4_18 & c4_19 < 14
3B200B	Did (s)he have fast breathing for 2 weeks or more?	a2_40 & a2_41 >= 14; c3_20 & c3_21 >= 14; c4_18 & c4_19 >= 14
3B210	Did (s)he have breathlessness?	not available
3B220A	Did (s)he have breathlessness for less than 2 weeks?	not available
3B220B	Did (s)he have breathlessness for 2 weeks or more?	not available
3B230	Was (s)he unable to carry out daily routines due to breathlessness?	a2_36 & a2_39_1 = "Walking/Exertion"
3B240	Was (s)he breathless while lying flat?	a2_36 & a2_39_1 = "Lying"

WHO 2012 VA code	WHO 2012 InterVA-4 indicator	Equivalent PHMRC variable(s) (IHME_PHMRC_VA_DATA_CODEBOOK_Y2013M09D11_0.xlsx)
3B250	Did you see the lower chest wall/ribs being pulled in as the child breathed?	c3_23; c4_20
3B260	Did (s)he have noisy breathing (grunting or wheezing)?	a2_42; c3_24; c4_23; c4_24
3B270	Did (s)he have severe chest pain?	a2_43
3B280	Did (s)he have diarrhoea?	a2_47; c3_44; c4_06
3B290A	Did (s)he have diarrhoea for less than 2 weeks?	a2_47 & a2_48 < 14; c4_06 & c4_08 < 14;
3B290B	Did (s)he have diarrhoea for 2 to 4 weeks?	a2_47 & a2_48 >= 14 & a2_48 < 28; c4_06 & c4_08 >=14 & c4_08 < 28
3B290C	Did (s)he have diarrhoea for 4 weeks or more?	a2_47 & a2_48 >= 28; c4_06 & c4_08 >= 28
3B300	At any time during the final illness was there blood in the stools?	a2_47 & a2_50; c4_11
3B310	Did (s)he vomit?	a2_53; c3_46
3B320	Did (s)he vomit "coffee grounds" or bright red/blood?	a2_55
3B330	Did (s)he have any abdominal problem?	a2_61; a2_64; a2_67; c4_40
3B340	Did (s)he have severe abdominal pain?	a2_61; a3_09
3B350A	Did (s)he have severe abdominal pain for less than 2 weeks?	a2_61 & a2_62 < 14
3B350B	Did (s)he have severe abdominal pain for 2 weeks or more?	a2_61 & a2_62 >= 14
3B360	Did (s)he have a more than usually protruding abdomen?	a2_64; c4_40
3B370A	Did (s)he have a more than usually protruding abdomen for less than 2 weeks?	a2_64 & a2_65 < 14
3B370B	Did (s)he have a more than usually protruding abdomen for 2 weeks or more?	a2_64 & a2_65 >= 14
3B380	Did (s)he have any mass in the abdomen?	a2_67
3B390A	Did (s)he have a lump inside the abdomen for less than 2 weeks?	a2_67 & a2_65 < 14
3B390B	Did (s)he have a lump inside the abdomen for 2 weeks or more?	a2_67 & a2_65 >= 14
3B400	Did (s)he have a severe headache?	a2_69
3B405	Did (s)he have a stiff or painful neck?	a2_72; c4_28
3B410A	Did (s)he have a stiff or painful neck for less than 1 week?	a2_72 & a2_73 < 7
3B410B	Did (s)he have a stiff or painful neck for 1 week or more?	a2_72 & a2_73 >= 7
3B420	Did (s)he have mental confusion?	a2_78
3B430A	Did (s)he have mental confusion for 3 months or more?	a2_78 & a2_79 > 3
3B440	Was (s)he unconscious for more than 24 hours before death?	a2_74 & a2_76 >=1; c4_27 = "24 hours or more"

WHO 2012 VA code	WHO 2012 InterVA-4 indicator	Equivalent PHMRC variable(s) (IHME_PHMRC_VA_DATA_CODEBOOK_Y2013M09D11_0.xlsx)
3B450	Did the unconsciousness start suddenly, quickly (at least within a single day)?	a2_74 & a2_76 >=1 & a2_75="Suddenly"
3B460	Did (s)he have convulsions?	a2_82; c3_25; c4_25
3B470A	Did the convulsions last for less than 10 minutes?	a2_82 & a2_83 < 0.007
3B470B	Did the convulsions last for 10 minutes or more?	a2_82 & a2_83 >= 0.007
3B480	Did (s)he become unconscious immediately after the convulsion?	a2_82 & a2_84
3B490	Did (s)he have any urine problems?	a2_52
3B500	Did (s)he pass no urine at all?	a2_52
3B510	Did (s)he go to urinate more often than usual?	not available
3B520	During the final illness did (s)he ever pass blood in the urine?	not available
3B530	Did (s)he have any skin problems?	a2_10; a2_13; a2_07; c4_30
3B540	Did (s)he have any ulcers, abscess or sores anywhere except on the feet?	a2_10
3B550	Did (s)he have any ulcers, abscess or sores on the feet that were not also on other parts of the body?	a2_13
3B560	During the illness that led to death, did (s)he have any skin rash?	a2_07; c4_30
3B570A	Did (s)he have the skin rash for less than 1 week?	a2_07 & a2_08 < 7; c4_30 & c4_33 < 7
3B570B	Did (s)he have the skin rash for 1 week or more?	a2_07 & a2_08 >= 7; c4_30 & c4_33 >=7
3B580	Did (s)he have measles rash?	not available
3B590	Did (s)he ever have shingles or herpes zoster?	not available
3B600	Did (s)he bleed from the nose, mouth or anus?	not available
3B610	Did (s)he have noticeable weight loss?	a2_18; c4_35
3B620	Was (s)he severely thin or wasted?	a2_18 & a2_19="Large"; c4_35
3B630	Did (s)he have mouth sores or white patches in the mouth or on the tongue?	c4_43
3B640	Did (s)he have stiffness of the whole body or was unable to open the mouth?	a2_87_7
3B650	Did (s)he have swelling (puffiness) of the face?	a2_25
3B660	Did (s)he have both feet swollen?	c4_36
3B670	Did (s)he have any lumps?	a2_29; a2_30; a3_01; a2_31
3B680	Did (s)he have any lumps or lesions in the mouth?	not available

WHO 2012 VA code	WHO 2012 InterVA-4 indicator	Equivalent PHMRC variable(s) (IHME_PHMRC_VA_DATA_CODEBOOK_Y2013M09D11_0.xlsx)
3B690	Did (s)he have any lumps on the neck?	a2_29
3B700	Did (s)he have any lumps on the armpit?	a2_30; c4_42
3B710	Did (s)he have any lumps on the groin?	a2_31
3B720	Did she have an ulcer or swelling in the breast?	a3_01
3B730	Did (s)he have paralysis of one side of the body?	a2_87_1; a2_87_2
3B740	Did (s)he have difficulty or pain while swallowing liquids?	a2_57
3B750	Did (s)he have yellow discoloration of the eyes?	a2_21
3B760	Did her/his hair colour change to reddish or yellowish?	c4_39
3B770	Did (s)he look pale (thinning/lack of blood) or have pale palms, eyes or nail beds?	a2_20; c4_41
3B780	Did (s)he have sunken eyes?	not available
3B790	Did (s)he drink a lot more water than usual?	not available
3B800	Did she have excessive vaginal bleeding in between menstrual periods?	a3_05
3B810	Did her vaginal bleeding stopped naturally during menopause?	a3_03
3B820	Did she have vaginal bleeding after menopause?	a3_04
3C100	Was she neither pregnant, nor delivered, within 6 weeks of her death?	NOT(a3_10 or a3_18 or a3_17)
3C110	Was she pregnant at the time of death?	a3_10
3C120	Did she die within 6 weeks of giving birth?	a3_18
3C130	Did she die within 6 weeks of a pregnancy that lasted less than 6 months?	a3_17
3C200	Did she die within 24 hours after delivery?	not available
3C210	Did she die during labour, but undelivered?	a3_15
3C220	Was she breastfeeding at death?	not available
3C230A	Was this the woman's first pregnancy?	not available
3C230B	Did she have four or more births, including stillbirths, before this pregnancy?	not available
3C240	Did she have any previous Caesarean section?	not available
3C250	Did she die during or after a multiple pregnancy?	not available
3C260	During pregnancy, did she suffer from high blood pressure?	not available

WHO 2012 VA code	WHO 2012 InterVA-4 indicator	Equivalent PHMRC variable(s) (IHME_PHMRC_VA_DATA_CODEBOOK_Y2013M09D11_0.xlsx)
3C270	Did she have foul smelling vaginal discharge during pregnancy or after delivery?	a3_20
3C280	During the last 3 months of pregnancy, did she suffer from convulsions?	not available
3C290	During the last 3 months of pregnancy did she suffer from blurred vision?	not available
3C300	Did she give birth to a live, healthy baby within 6 weeks of death?	not available
3C310	Was there excessive vaginal bleeding during pregnancy or after delivery?	a3_13; a3_14; a3_19
3C320	Was there vaginal bleeding during the first 6 months of pregnancy?	not available
3C330	Was there vaginal bleeding during the last 3 months of pregnancy but before labour started?	a3_13
3C340	Was there excessive vaginal bleeding during labour?	a3_14
3C350	Was there excessive vaginal bleeding after delivering the baby?	a3_19
3C360	Was the placenta not completely delivered?	not available
3C365	Did she deliver or try to deliver an abnormally positioned baby?	not available
3C370	Was she in labour for unusually long (more than 24 hours)?	a3_16 > 1
3C380	Did she attempt to terminate the pregnancy?	not available
3C390	Did she recently have a pregnancy that ended in an abortion (spontaneous or induced)?	a3_17
3C400	Did she give birth in a health facility?	not available
3C410	Did she give birth at home?	not available
3C420	Did she give birth elsewhere, e.g. on the way to a facility?	not available
3C430	Did she receive professional assistance during the delivery?	not available
3C440	Did she have an operation to remove her uterus shortly before death?	not available
3C450	Did she have a normal vaginal delivery?	not available
3C460	Did she have an assisted delivery, with forceps/vacuum?	not available
3C470	Did she deliver by Caesarean section?	not available
3C480	Was the baby born more than one month early?	not available

WHO 2012 VA code	WHO 2012 InterVA-4 indicator	Equivalent PHMRC variable(s) (IHME_PHMRC_VA_DATA_CODEBOOK_Y2013M09D11_0.xlsx)
3D100	Was the child part of a multiple birth?	c1_01= "Multiple"
3D110	Was the child born in a complicated delivery?	c2_01_5; c2_01_6; c2_01_14; c2_10 >= 1 & c2_10 < 7
3D120	Was the baby born 24 hours or more after the water broke?	c2_07 = "One day or more"
3D130	Did the baby stop moving in the womb before labour started?	c2_04 = "No"
3D140	Was baby born in a normal vaginal delivery?	c2_17 = "Vaginal Don't Know"; c2_17 = "Vaginal without Forceps"
3D150	Was baby born with forceps/vacuum?	c2_17 = "Vaginal with Forceps"
3D160	Was baby delivered by Caesarean section?	c2_17 = "C-section"
3D170	Did the baby's bottom, feet, arm or hand come into the vagina before its head?	c2_01_5
3D180	Was the baby of abnormal size?	c1_08b < 2500; c1_08b > 4500
3D190	Was the baby smaller than normal, weighing under 2.5 kg?	c1_08b < 2500
3D200	Was the baby larger than normal, weighing over 4.5 kg?	c1_08b > 4500
3D210A	Had the pregnancy lasted less than 34 weeks when the baby was born?	c2_02 = 210; c2_03 = "Early"
3D210B	Had the pregnancy lasted between 34 and 37 weeks when the baby was born?	c2_02 = 240
3D210C	Had the pregnancy lasted more than 37 weeks when the baby was born?	c2_02 = 270; c2_03 = "On time"; c2_03 = "Late"
3D220	Was the umbilical cord wrapped several times (more than once) around the neck of the child at birth?	c2_01_7
3D230	Did the baby/the child have any noticeable malformation?	c3_02
3D240	Did the baby/the child have a swelling/defect on the back?	c3_03_3
3D250	Did the baby/the child have a very large head?	c3_03_2
3D260	Did the baby/the child have a very small head?	c3_03_1
3D270	Was the baby/the child not growing normally?	not available
3D280	Was the baby blue in colour at birth?	not available
3D290	Did the baby cry after birth, even if only a little bit?	c3_07
3D300	Did the baby breathe after birth, even a little?	c3_04
3D310	Was the baby given assistance to breathe at birth?	c3_06
3D320	If the baby did not cry or breathe, was it born dead?	c1_11 = "Dead"; c1_26 = "Stillbirth"; c1_15

WHO 2012 VA code	WHO 2012 InterVA-4 indicator	Equivalent PHMRC variable(s) (IHME_PHMRC_VA_DATA_CODEBOOK_Y2013M09D11_0.xlsx)
3D330	Was the dead baby macerated, that is, showed signs of decay?	c1_17
3D340	Was the baby able to suckle or bottle-feed within the first 24 hours after birth?	c3_11
3D350	Did the baby stop suckling or bottle feeding 3 days after birth?	c3_14 >= 3
3D360	Did the baby have convulsions starting within the first day of life?	not available
3D370	Did the baby have convulsions starting on the second day or later after birth?	not available
3D380	Did the baby's body become stiff, with the back arched backwards?	not available
3D390	Did the child have a bulging or raised fontanelle?	c3_24
3D400	Did the child have a sunken fontanelle?	not available
3D410	Did the baby become unresponsive or unconscious soon after birth (within less than 24 hours)?	not available
3D420	Did the baby become unresponsive or unconscious more than 1 day after birth?	c3_33
3D430	Did the baby become cold to the touch before it died?	c3_29
3D440	Did the baby have redness or discharge from the umbilical cord stump?	c3_35; c3_36
3D450	Did the baby have yellow palms or soles?	c3_47; c3_48
3D460	Did the mother NOT receive tetanus toxoid (TT) vaccine?	c2_11 = "No"
3D500A	Was this baby born from the mother's first pregnancy?	c1_02 = "First"
3D500B	Did the mother have 4 or more births before this baby was born?	not available
3D510	During pregnancy, did the baby's mother suffer from high blood pressure?	c2_01_2
3D520	Did the baby's mother have foul smelling vaginal discharge during pregnancy or after delivery?	not available
3D530	During the last 3 months of pregnancy, did the baby's mother suffer from convulsions?	c2_01_1
3D540	During the last 3 months of pregnancy did the baby's mother suffer from blurred vision?	not available

WHO 2012 VA code	WHO 2012 InterVA-4 indicator	Equivalent PHMRC variable(s) (IHME_PHMRC_VA_DATA_CODEBOOK_Y2013M09D11_0.xlsx)
3D550	Did the baby's mother have vaginal bleeding during the last 3 months of pregnancy but before labour started?	c2_01_8
3D560	Was the child born in a health facility?	c2_13a = "Hospital"; c2_13a = "Other Heath Facility"
3D570	Was the child born at home?	c2_13a = "Home"
3D580	Was the child born somewhere else, e.g. on the way to a facility?	c2_13a = "On Route to Health Facility"
3D590	Did the mother receive professional assistance during the delivery?	c2_15a = "Doctor"; c2_15a = "Nurse/midwife"
3E100	Did (s)he suffer from any injury or accident that led to her/his death?	a5_01_1; a5_01_2; a5_01_3; a5_01_4; a5_01_5; a5_01_6; a5_01_7; a5_01_9a; c4_47_1; c4_47_2; c4_47_3; c4_47_4; c4_47_5; c4_47_6; c4_47_7; c4_47_8a
3E110	Was it a road traffic accident?	a5_01_1; c4_47_1
3E300	Was (s)he Injured in a non-road transport accident?	not available
3E310	Was (s)he Injured in a fall?	a5_01_2; c4_47_2
3E320	Did (s)he die of drowning?	a5_01_3; c4_47_3
3E330	Did (s)he suffer from burns?	a5_01_6; c4_47_6
3E340	Did (s)he suffer from any plant/ animal/insect bite or sting that led to her/his death?	a5_01_5; c4_47_5
3E500	Was (s)he injured by a force of nature?	not available
3E510	Was there any poisoning?	a5_01_4; c4_47_4
3E520	Was (s)he subject to violence/assault?	a5_01_7; c4_47_7
3E530	Was the injury intentionally inflicted by someone else?	a5_03; c4_48
3E700	Do you think (s)he committed suicide?	a5_02
3F100	Did (s)he drink alcohol?	a4_05
3F110	Did (s)he smoke tobacco (cigarette, cigar, pipe, etc.)?	a4_01
3G100	Was (s)he adequately vaccinated?	not available
3G110	Did (s)he receive any treatment for the illness that led to death?	a6_01
3G120	Did (s)he receive oral rehydration salts?	not available
3G130	Did (s)he receive (or need) intravenous fluids (drip) treatment?	not available
3G140	Did (s)he receive (or need) a blood transfusion?	not available
3G150	Did (s)he receive (or need) treatment/food through a tube passed through the nose?	not available

WHO 2012 VA code	WHO 2012 InterVA-4 indicator	Equivalent PHMRC variable(s) (IHME_PHMRC_VA_DATA_CODEBOOK_Y2013M09D11_0.xlsx)
3G160	Did (s)he receive (or need) injectable antibiotics?	not available
3G170	Did (s)he have (or need) an operation for the illness?	not available
3G180	Did (s)he have the operation within 1 month before death?	not available
3G190	Was (s)he discharged from hospital very ill?	not available
4A100	In the final days before death, did (s)he travel to a hospital or health facility?	not available
4A110	Did (s)he use motorised transport to get to the hospital or health facility?	not available
4A120	Were there any problems during admission to the hospital or health facility?	not available
4A130	Were there any problems with the way (s)he was treated (medical treatment, procedures, inter-personal attitudes, respect, dignity) in the hospital or health facility?	not available
4A140	Were there any problems getting medications, or diagnostic tests in the hospital or health facility?	not available
4A150	Does it take more than 2 hours to get to the nearest hospital or health facility from the deceased's household?	not available
4A160	In the final days before death, were there any doubts about whether medical care was needed?	not available
4A170	In the final days before death, was traditional medicine used?	not available
4A180	In the final days before death, did anyone use a telephone or cell phone to call for help?	not available
4A190	Over the course of illness, did the total costs of care and treatment prohibit other household payments?	not available
